# Electroacupuncture Potentiates Cannabinoid Receptor-Mediated Descending Inhibitory Control in a Mouse Model of Knee Osteoarthritis

**DOI:** 10.3389/fnmol.2018.00112

**Published:** 2018-04-06

**Authors:** Xiao-Cui Yuan, Bing Zhu, Xiang-Hong Jing, Li-Ze Xiong, Cai-Hua Wu, Fang Gao, Hong-Ping Li, Hong-Chun Xiang, He Zhu, Bin Zhou, Wei He, Chuan-You Lin, Hui-Lin Pan, Qiang Wang, Man Li

**Affiliations:** ^1^Department of Neurobiology and Key Laboratory of Neurological Diseases of Ministry of Education, The Institute of Brain Research, School of Basic Medicine, Tongji Medical College, Huazhong University of Science and Technology, Wuhan, China; ^2^Institute of Acupuncture and Moxibustion, China Academy of Chinese Medical Sciences, Beijing, China; ^3^Department of Anesthesiology, Xijing Hospital, Fourth Military Medical University, Xi’an, China; ^4^Department of Anesthesiology and Perioperative Medicine, The University of Texas MD Anderson Cancer Center, Houston, TX, United States; ^5^Department of Anesthesiology, First Affiliated Hospital of Xi’an JiaoTong University, Xi’an, China

**Keywords:** knee osteoarthritis (KOA), electroacupuncture analgesia, diffuse noxious inhibitory controls (DNIC), endocannabinoids, chronic pain

## Abstract

Knee osteoarthritis (KOA) is a highly prevalent, chronic joint disorder, which can lead to chronic pain. Although electroacupuncture (EA) is effective in relieving chronic pain in the clinic, the involved mechanisms remain unclear. Reduced diffuse noxius inhibitory controls (DNIC) function is associated with chronic pain and may be related to the action of endocannabinoids. In the present study, we determined whether EA may potentiate cannabinoid receptor-mediated descending inhibitory control and inhibit chronic pain in a mouse model of KOA. We found that the optimized parameters of EA inhibiting chronic pain were the low frequency and high intensity (2 Hz + 1 mA). EA reversed the reduced expression of CB1 receptors and the 2-arachidonoylglycerol (2-AG) level in the midbrain in chronic pain. Microinjection of the CB1 receptor antagonist AM251 into the ventrolateral periaqueductal gray (vlPAG) can reversed the EA effect on pain hypersensitivity and DNIC function. In addition, CB1 receptors on GABAergic but not glutamatergic neurons are involved in the EA effect on DNIC function and descending inhibitory control of 5-HT in the medulla, thus inhibiting chronic pain. Our data suggest that endocannabinoid (2-AG)-CB1R-GABA-5-HT may be a novel signaling pathway involved in the effect of EA improving DNIC function and inhibiting chronic pain.

## Introduction

Chronic pain is often persistent and poorly treated by existing therapies in clinic, which is an important focus in research of mechanism of pain and acupuncture analgesia (Marchand et al., 2005; Zhang et al., 2014). It has been proved that electroacupuncture (EA) is effective in relieving chronic pain in patients with knee osteoarthritis (KOA; Weiner et al., 2013). However, the involved mechanisms remain unclear.

The endocannabinoid system is an important neuromodulatory system involved in control of pain transmission within central nervous system. Both ligands and receptors can be detected in the periphery, at the level of the spinal cord, and in nociceptive regions of brain (Hu et al., 2014). We have shown that EA significantly increases the anandamide level and activates CB2 receptors in inflammatory skin tissues, thus attenuating acute inflammatory pain (Chen et al., 2009; Zhang et al., 2010). EA also reduces acute inflammatory pain through activation of CB1 receptors in the striatum (Nishihara et al., 2013). Chronic pain is a major clinical symptom in patients with KOA and typically worsens with weight bearing and movement of the affected joint (Hart, 1974; Ferreira-Gomes et al., 2012). EA has been shown to be effective in the treatment of chronic pain of KOA in randomized controlled trials (RCT; Plaster et al., 2014; Ding et al., 2016). We wonder whether EA may potentiate cannabinoid receptor mediated descending inhibitory control and inhibit chronic pain in a mouse model of KOA.

Previous psychophysical studies suggested a decrease of the so-called pain-inhibiting-pain effect, named diffuse noxious inhibitory controls (DNIC) in chronic pain patients (Quante et al., 2008), which occurs when response from a painful stimulus is inhibited by another, often spatially distant, noxious stimulus (Le Bars, 2002). The strength of DNIC may reflect neuronal plasticity of the descending pain inhibitory system, and DNIC is often diminished during the development of chronic pain (Quante et al., 2008). The periaqueductal gray (PAG) and rostral ventromedial medulla (RVM) are major brain regions involved in the descending pain control, both opioids and cannabinoids may produce their effects by reducing GABAergic inhibition of serotonin neurons in the PAG and RVM (Osborne et al., 1996; Finn et al., 2003).

Since blockade of opioid receptors prevents analgesia produced by DNIC function in rats with muscle inflammation (de Resende et al., 2011), the deficit of endocannabinoids may contribute to the impaired DNIC function of chronic pain. Therefore, in the present study, we first evaluated the changes of DNIC function and screen the best parameters of EA which can reverse the imparied DNIC function and inhibit chronic pain in a mouse model of KOA. Then we determined whether endocannabinoid ligands and CB receptors were involved in the mechanism of EA improving DNIC function and inhibiting chronic pain. Using conditioning knock mice, we also determined whether CB1 receptors on GABAergic or glutamatergic neurons were involved in the effect of EA on pain hypersensitivity and DNIC function. Finally, we determined whether CB1-GABA pathway underlied the effects of EA on descending inhibitory control of 5-HT, thus increasing DNIC function and inhibiting chronic pain.

## Materials and Methods

### Animals

All animal experiments were approved by the Animal Care and Use Committee at Huazhong University of Science and Technology and conformed to the ethical guidelines of the International Association for the Study of Pain (Zimmermann, 1983). Eight-weeks-old female C57BL/6 mice were obtained from Experimental Animal Center of Tongji Medical College of Huazhong University of Science and Technology. Mice were randomly divided into different groups by using SPSS 17.0 software.

We crossed CB1R-floxed mice with Gad2-icre ERT mice to obtain the GABA-CB1R-KO mouse line and crossed with Vglut1-icre ERT mice to obtain the Glu-CB1R-KO mouse line. Deletion of CB1R gene from GABA-CB1R-KO and Glu-CB1R-KO mice obtained in mice by eight daily injections of tamoxifen (Sigma, St. Louis, MO, USA; 1 mg, intraperitoneal (i.p.); Han et al., 2012). Mutant mice were kindly provided by Dr. Lize Xiong (Fourth Military Medical University, Xi’an, China). The mice were individually housed in cages with a 12-h light/dark cycle and had free access to food and water. The transgenic mice and wild type mice had no significant difference in phenotype.

### Induction of Knee Osteoarthritis (KOA)

KOA was induced by intra-articular injection of monosodium iodoacetate (MIA; Sigma, UK) into the left knee joint after mice were briefly anesthetized with 10% chloralic hydras. The knee joint was shaved and flexed at a 90° angle. Five microliters of 5 mg/ml MIA in sterile saline (0.9%) were injected through the infrapatellar ligament into the joint space of the left knee with a 30-gauge needle (La Porta et al., 2013). This concentration of MIA causes histological changes in the cartilage (van Osch et al., 1994) and induces joint pain (Harvey and Dickenson, 2009) in mice. Control mice received an intra-articular injection of vehicle (5 μl of sterile saline, 0.9%).

### EA Treatment

The animals were habituated to the restricting bag for 3 days before KOA induction, 30 min each day. In the EA treatment group, mice received EA administration on the left “Neixiyan” (Ex-LE4) and “Dubi” (ST35) once every other day for 4 weeks, starting from 2 days after MIA injection. EA (1 mA /0.1 mA and 0.1 ms) was administered at the different frequency (2 Hz, 15 Hz or 100 Hz) for 30 min. The EA treatment and behavioral analysis is not the same day. The mice received EA administration in the even-numbered days after MIA injection, and measure behavior tests in the odd-numbered days after MIA injection. Current was delivered with a Han’s Acupoint Nerve Stimulator (LH202, Huawei Co. Ltd., Beijing, China). Two acupuncture needles were inserted into two acupoints corresponding to Ex-LE4 and ST35 in humans. Ex-LE4 is located at the medial cavity of the patella and the patellar ligament, and ST35 lies on the lateral cavity of the patella and patellar ligament. A schematic diagram is presented in Supplementary Figure S1. Ex-LE4 and ST35 were chosen, because their using frequency is the highest in KOA and they are specific acupoints for treating knee problems (Ng et al., 2003; Selfe and Taylor, 2008).

### Nociceptive Behavioral Tests

Mechanical allodynia and heat hyperalgesia were also demonstrated in the hind paw of animals with KOA, using von Frey filaments and the hot plate test, respectively (Fernihough et al., 2004; La Porta et al., 2013). The behavioral tests were performed three times before KOA induction and once every other day, starting from the first day to 4 weeks after KOA induction. The animals were habituated to the testing environment for 30 min.

The hot plate test was used to measure the response latencies according to a previously described method (Ferreira et al., 1999). A glass cylinder (40 cm high, 20 cm diameter) was used to keep mice on the hot surface of the plate, which was maintained at a temperature of 55 ± 0.5°C. The time (s) between placement of the mouse and the shaking or licking of paws or jumping was recorded as the index of response latency. A latency period (cut-off) of 30 s was defined as complete analgesia (Pigatto et al., 2017). The test was repeated three times in mice, and the mean value was calculated.

Mechanical allodynia was assessed by placing mice on an elevated mesh floor, and the tactile threshold was measured by using the “up-down” method (Chaplan et al., 1994; La Porta et al., 2013). After an acclimation period of 30 min, a series of calibrated von Frey filaments (Stoelting, Kiel, WI, USA) were applied perpendicularly to the plantar surface of the left hindpaw with sufficient force to bend the filament for 6 s. Brief withdrawal or paw flinching was considered as a positive response. The test was repeated two to three times in mice, and the mean value was calculated.

### DNIC Function Tests

The mice were habituated to the procedure of DNIC function tests for 3 days to avoid acute stress.The mechanical allodynia threshold of left hindpaw was measured by von Frey filaments as M1. Then the tail of the mouse was immersed into hot water (48 ± 0.5°C) for 1 min as a noxious conditioning stimulus, and the mechanical allodynia threshold of left hindpaw was immediately measured again as M2 (Figure 2E). DNIC function was assessed by measuring the difference in mechanical allodynia threshold before and after tail immersion (DNIC% = (M2 − M1)/M1 * %). Thus, normal DNIC function is reflected by the increased ratio in mechanical allodynia threshold by the conditioning hot noxious stimulation (Landau et al., 2010).

**Figure 1:**
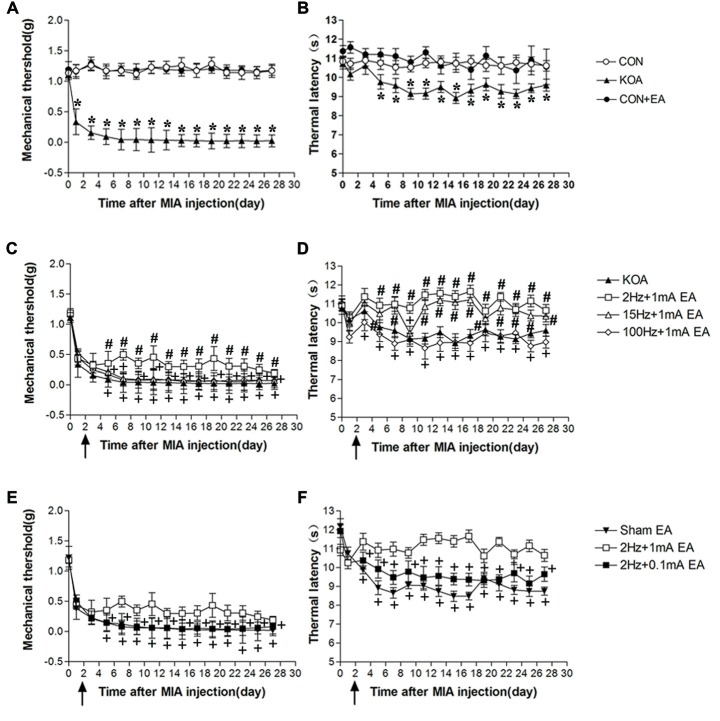
Time course of the effect of electroacupuncture (EA) on pain hypersensitivity in knee osteoarthritis (KOA) mice. **(A,B)** Time course of tactile threshold in response to von Frey filaments **(A)** or a noxious heat stimulus **(B)** in control, control+EA and KOA mice. **(C,D)** Time course of the effect of different EA parameters on tactile **(C)** and thermal **(D)** withdrawal thresholds of KOA mice. **(E,F)** Time course of the effect of EA on tactile **(E)** and thermal **(F)** withdrawal thresholds of KOA mice. EA was administered for 30 min, once every other day for 4 weeks, starting from 2 days after monosodium iodoacetate (MIA) injection, as indicated by arrows. Data are expressed as means ± SD (*n* = 10 mice in each group). *p < 0.05, compared with the control group; ^#^*p* < 0.05, compared with the KOA group; ^+^*p* < 0.05, compared with the 2 Hz + 1 mA group.

**Figure 2:**
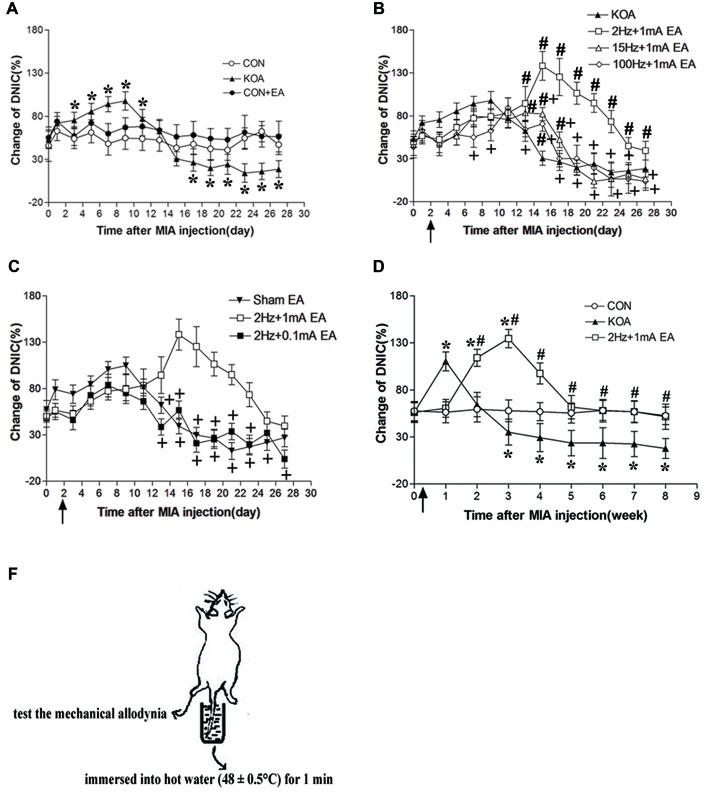
Changes in the diffuse noxius inhibitory controls (DNIC) function and EA effect during chronic pain development after KOA induction. **(A)** Time course of changes in DNIC function in control, control+EA and KOA mice. **(B)** Time course of the EA effect on DNIC function in KOA mice. **(C)** Effect of EA on DNIC function in KOA mice. **(D)** Time course of the effect of 2 Hz + 1 mA EA on DNIC function in KOA mice. **(E)** Graphic drawing show test used for assessing the DNIC function in mice. EA was administered for 30 min, once every other day for 4 or 8 weeks starting from 2 days after MIA injection, as indicated by arrows. Data are expressed as means ± SD (*n* = 10 mice in each group). *p < 0.05, compared with the control group; ^#^*p* < 0.05, compared with the KOA group; ^+^*p* < 0.05, compared with the 2 Hz + 1 mA group.

### HPLC-MS-MS Quantification of Anandamide and 2-Arachidonoylglycerol

N-arachidonoylethanolamine (AEA) and 2-arachidonoylglycerol (2-AG) were purchased from Sigma (Milwaukee, WI, USA), and AEA-d8 was purchased from Cayman Chemicals (Ann Arbor, MI, USA). AEA-d8 was used as an internal standard (IS). The purity of the reference compounds was ≥98%.

The midbrain tissues were weighed and placed into borosilicate glass culture tubes containing 2 ml of acetonitrile with AEA-d8 at a concentration of 5 μg/ml. Tissues were homogenized with a glass rod and sonicated in water for 1 h followed by incubation overnight at –20°C to precipitate proteins. After centrifugation at 1,500 *g* for 10 min, the supernatant was removed and evaporated to dryness under nitrogen gas, resuspended in 500 μl methanol, and dried again. The final extract was resuspended in 50 μl methanol (Patel et al., 2003).

A series Surveyor^®^ MS pump with Surveyor autosampler from Thermo Fisher Scientific (San Jose, CA, USA), was used for the chromatographic run. The HPLC system was coupled to a Ion Trap mass spectrometer, equipped with a positive electrospray ionization (ESI) source. The analytes were separated using a reversed phase C18 100 × 2.10 mm from (Thermo Fisher Scientific, San Jose, CA, USA), packed with 5 μm average diameter core-shell particles. A guard column was also used to protect the HPLC system. The mobile phases were water (phase A) and 2.5 mM formic acid in methanol (phase B), at a flow rate of 0.2 ml/min. The following gradient elution scheme was used: increase of the organic phase from 80% to 84% in 3 min and linearly to 100% in the following 0.5 min. Finally, after 3 min of 100% B, the column was led to the original ratio of 20% A and 80% B within 8 min to enable equilibration of the column (Sergi et al., 2013).

### Quantitative Polymerase Chain Reaction

The midbrain tissues were removed 4 weeks after vehicle or MIA injection. The tissues were excised from mice immediately after the animals were anesthetized with 10% chloralic hydras and decapitated. Total RNA was isolated from the midbrain specimens by using Trizol reagent (Invitrogen, USA). Aliquots of 2 μg total RNA were reverse transcribed into cDNA using ReverTra Ace-a-TM (Toyobo, Japan). The PCR mixture (10 μl) consisted of 1 μl diluted cDNA, 5 μl SYBR quantitative polymerase chain reaction (qPCR) mix (Toyobo, Japan), 1 μl of each primer, and 3 μl water. qPCR was performed using CFX96 system (Bio-Rad, UK), and the relative expression levels were quantified with CFX Manager software. The expression level of each gene was determined by the threshold cycle (CT). The amount of target mRNAs, normalized to the endogenous control (β-actin), was obtained by 2^−ΔΔCT^. Results of independent experiments were expressed as the percentage change over mRNA level of the control group. Specific sequence primers are listed in Table 1 (Tam et al., 2010).

**Table 1:** List of primers used for quantitative polymerase chain reaction (qPCR).

Gene names	Primers
Mouse CB1	Sense, 5′-GTACCATCACCACAGACCTCCTC-3′
	Antisense, 5′-GGATTCAGAATCATGAAGCACTCCA-3′
Mouse CB2	Sense, 5′-GGCTGACAAATGACACCCAGT-3′
	Antisense, 5′-CACTTCTGTCTCCCGGCATC-3′
Mouse β-actin	Sense, 5′-CCCAGACATCAGGGAGTAATGG-3′
	Antisense, 5′-TCTATCGGATACTTCAGCGTCA-3′

### Western Blotting

The midbrain and medulla and spinal cord tissues were removed as described above, minced with scissors, and homogenized in RIPA lysis buffer (40 mg/ml for tissues, Beyotime Biotechnology, Nanjing, China) and 2 mM phenylmethylsulfonyl fluoride and centrifuged at 12,000 *g* for 15 min. The pellet was discarded, and protein concentrations of the supernatant were determined by using the Enhanced BCA Protein Assay Kit (Beyotime Biotechnology, China). Proteins were denatured with sodium dodecyl sulfatepolyacrylamide gel electrophoresis (SDS-PAGE) loading buffer at 95°C for 5 min and separated on a 10% glycine-SDS-PAGE gel. The proteins were transferred onto a polyvinylidene fluoride membrane, blocked for 1 h by 5% non-fat dry milk in Tris-buffered saline (TBS) containing 0.1% Tween-20. The membrane was incubated with goat anti-CB1 antibody (1:500, Santa Cruz, Dallas, TX, USA); rabbit anti-CB2 antibody(1:1000, Abcam, Hong Kong); or mouse anti-β-actin antibody (1:5000; Santa Cruz, Dallas, TX, USA) at 4°C overnight. After three times washes in 0.1% TBS-Tween 20 (pH, 7.4), the membranes were then incubated with horseradish peroxidase-conjugated secondary antibodies from Santa Cruz Biotechnology: rabbit anti-goat secondary antibody (1:20,000), goat anti-rabbit secondary antibody (1:20,000) or goat anti-mouse secondary antibody (1:20,000) for 1 h at room temperature and washed three times. The enhanced chemiluminescence method (ECL Plus Western blotting detection reagents, Pierce, Rockford, IL, USA) was used to reveal the protein bands according to the manufacturer’s protocol. The optical density of each band was then measured with a computer-assisted imaging analysis system (Quantity One, Bio-Rad, UK) and normalized with β-actin. Results of independent experiments were expressed as the percentage change over the protein amount in the control group.

### Double-Immunofluorescence Labeling

Mice were deeply anesthetized with 10% chloralic hydras and were transcardially perfused with 37°C normal saline followed by 4% paraformaldehyde in 0.1 M phosphate buffer (pH 7.4; 4°C). The brain were quickly removed and post fixed for 4 h in the same fixative solution and cryoprotected in 30% sucrose in 0.1 M phosphate buffer for 48 h at 4°C. The sections were cut at 20 μm on a cryostat, which were mounted onto gelatin-coated slides and air dried overnight.

The sections were rinsed in 0.01 M PBS and blocked for 1 h with 5% donkey serum and 0.2% tween-20 in PBS and then incubated with the following primary antibodies at 37°C for 1 h and at 4°C overnight: rabbit anti-CB1 (1:400, Abcam, Hong Kong); mouse anti-GAD67 (1:100, Abcam, Hong Kong); guinea pig anti-VGLUT2 (1:100, Millipore, USA). Subsequently, sections were washed four times in PBS for 5 min and incubated with corresponding secondary antibodies from Jackson Immunoresearch (West Grove, PA, USA): donkey anti-rabbit IgG conjugated with Dylight594 (1:400); donkey anti-mouse IgG conjugated with Dylight 488 (1:400) and donkey anti-guinea pig IgG conjugated with Dylight 488 (1:400). Sections were washed four times in PBS for 5 min and then coverslipped. Negative controls were included by omitting the primary antibodies and with primary antibodies preabsorbed with their specific blocking peptides in the above procedures, which resulted in no positive labeling in the brain tissues. For quantification, four to five brain sections containing the PAG were randomly selected from each mouse and digital images were captured using an LSM780 confocal laser scanning fluorescent microscope system (ZSSIS, Germany). All images for each experiment were taken at the same time with the same camera settings, and the authors performing the image analysis were blinded to group. Positive immunostaining for GAD67, or VGLUT2 was defined using a constant threshold for each protein applied ventrolateral periaqueductal gray (vlPAG) from four to five sections per mouse. CB1 with GAD67, or VGLUT2 immunostaining were counted in the vlPAG from four to five sections per mouse. Results are reported as % of the area CB1 with GAD67, or VGLUT2 immunostaining in the area of total GAD67 or VGLUT2.

### Drug Administration

AM251[1-(2,4-dichlorophenyl)5-(4-iodophenyl)-4-methyl-N-1-piperidinyl-1H-pyrazole-3-carboxamide], as a CB1 receptor antagonist (Sigma-Aldrich, Steinheim, Germany), were dissolved in the dimethyl sulfoxide (DMSO; Sigma-Aldrich) to achieve a concentration of 10 mg/ml, and, on the day of the experiment, 10 μl were dissolved in 100 μl of DMSO to achieve a final concentration of 1 mg/ml (Tjen et al., 2009).

The baseline nociceptive thresholds and DNIC function were tested for 3 days before MIA injection, and the mean value was calculated as baseline data. Mice were randomly divided into CON+DMSO, KOA+DMSO, EA+AM251 and EA+DMSO groups. Since the efficacy of AM251 is dose-dependent, we chose the dose of the CB1 receptor antagonist AM251 based on previous study (Esmaeili et al., 2016). On the 14th day after KOA induction, each mouse in the EA+AM251 group was microinjection with of AM251 0.1 μl into the vlPAG. EA was administered for 30 min, after 2.5 h AM251 microinjection (Tjen et al., 2009). Others groups received an equal volume of DMSO microinjection. Treatments were randomly allocated to animals and the observer was unaware of treatment allocations.

### Intra-vlPAG Microinjection

Mice were anesthetized with with 10% chloralic hydras (3.5 kg/ml, intraperitoneal) and implanted with a 10 mm-long guide cannula 0.5 mm above the right vlPAG (AP: −4.8 mm, LM: −0.5 mm from midline, DV: −2.8 mm, from the skull surface) according to a mouse atlas (Dunham et al., 1993). After cannulation, animals were returned to the holding room for at least 7 days to recover from the surgery.

On the 14th day after KOA induction, a 30-gauge injection cannula, connected to a 1 μl Hamilton syringe, was extended 0.5 mm beyond the tip of guide cannula for injecting the drug solution into the vlPAG. A microinfusion pump (KDS311, KD Scientific Inc.) was used to deliver the drug solution of 0.1 μl over 1 min. The injection cannula was left at the injection site for an additional 5 min to allow for complete diffusion of the injected drug (Lee et al., 2016).

### Measurements of Serotonin (5-HT)

After sacrificing the mouse, medulla tissue was immediately removed. After weighing, specimens were homogenized in 3 ml of acid butyl alcohol and centrifuged at 3000 *g* for 5 min before collecting the supernatant. The supernatant (2.5 ml) was added 5 ml n-heptane and 1 ml 0.1 N HCl, vortexed for 5 min, and centrifuged at 3000 *g* for 5 min at room temperature. The water phase (0.6 ml) was collected and mixed with 0.12 ml of 0.5% cysteine and 3 ml of 0.006% o-phenyl-diformaldehyde in 10 N HCl. After mixing thoroughly, the solution was placed into boiling water for 10 min. The fluorescence intensity of 5-HT was measured at 365/460 nm by F-4500 Flourescence Spectrophotometer (Hitachi, Japan) and 5-HT concentration was determined by comparing to a standard 5-HT dilution (Li et al., 2003).

### Data Analysis

Data are presented as means ± SD. To determine the statistical difference in the withdrawal thresholds, we used two-way repeated-measures ANOVA where each factor as a “between-subjects” (group) or “repeated measures factor (time points), and Bonferroni’s *post hoc* test was used for multiple comparisons using SPSS version 17.0 software. Other data were analyzed by one-way ANOVA followed by Newman-Keuls* post hoc* test with two-tailed hypothesis. A *P* value less than 0.05 adjusted by SPSS version 17.0 software was considered significant”.

## Results

### EA at 2 Hz + 1 mA Most Effectively Reduces Pain Hypersensitivity in KOA Mice

Eight groups of mice (control, control+EA, KOA, 2 Hz + 0.1 mA EA, 2 Hz + 1 mA EA, 15 Hz + 1 mA EA, 100 Hz + 1 mA EA, and sham EA) were used for this series of experiments. KOA induction significantly reduced mechanical withdrawal threshold and thermal withdrawal latency (Figures 1A,B).

At 2 Hz + 1 mA, EA significantly increased the thermal withdrawal latency and tactile threshold in KOA mice (Figures 1C,D). At 15 Hz + 1 mA, EA had no significant effect on mechanical allodynia (Figure 1C), but significantly increased the thermal withdrawal latency in KOA mice (Figure 1D). EA at 100 Hz + 1 mA had no significant effect on the thermal latency and tactile threshold in KOA mice (Figures 1C,D). Moreover, 2 Hz + 1 mA EA had no significant effect on mechanical allodynia and thermal hyperalgesia of control mice (Figures 1A,B). These data indicate that the optimal EA frequency is 2 Hz for achieving the most analgesic effect in this KOA model.

EA at 2 Hz + 0.1 mA had no significant effect on thermal hyperalgesia and mechanical allodynia of KOA mice (Figures 1E,F). We thus used 1 mA to evaluate the EA effect in the subsequent experiments.

### EA at 2 Hz + 1 mA Most Effectively Improves Reduced DNIC Function of KOA Mice

DNIC function in patients with KOA is gradually decreased with the development of chronic pain (Quante et al., 2008). Three days after KOA induction, the DNIC function was significantly increased compared with the baseline value and maintain to 11 days after KOA induction (Figure 2A). However, 17 days after KOA induction, DNIC function was significantly reduced (Figure 2A).

Compared with the KOA group, EA at 2 Hz + 1 mA, EA significantly increased the DNIC function 13 days after KOA induction (Figure 2B). In addition, the effect of EA at 2 Hz + 1 mA group on the DNIC function was maintained to 8 weeks after KOA induction (Figure 2D). At 15 Hz + 1 mA, EA also significantly increased the DNIC function 13–17 days after KOA induction (Figure 2B). At 100 Hz + 1 mA, EA increased the DNIC function only at 15 days after KOA induction (Figure 2B). The EA effect at 15 Hz + 1 mA and 100 Hz + 1 mA groups on the DNIC function was significantly lower than that of 2 Hz + 1 mA group (Figure 2B). Also, the EA effect at 2 Hz + 0.1 mA on the DNIC function was significantly lower than that produced by 2 Hz + 1 mA EA (Figure 2C). Moreover, 2 Hz + 1 mA EA had no significant effect on the DNIC function of control mice (Figure 2A). Thus, EA at 2 Hz + 1 mA is most effective in improving DNIC function of KOA mice.

Since KOA and sham EA group had no significant difference in behavioral and DNIC function tests, so in the following experiment three groups of mice were used: control, KOA, 2 Hz + 1 mA EA.

### EA Reverses the Reduction of CB1 but Not CB2 Receptor Expression in the Midbrain of KOA Mice

**Figure 3:**
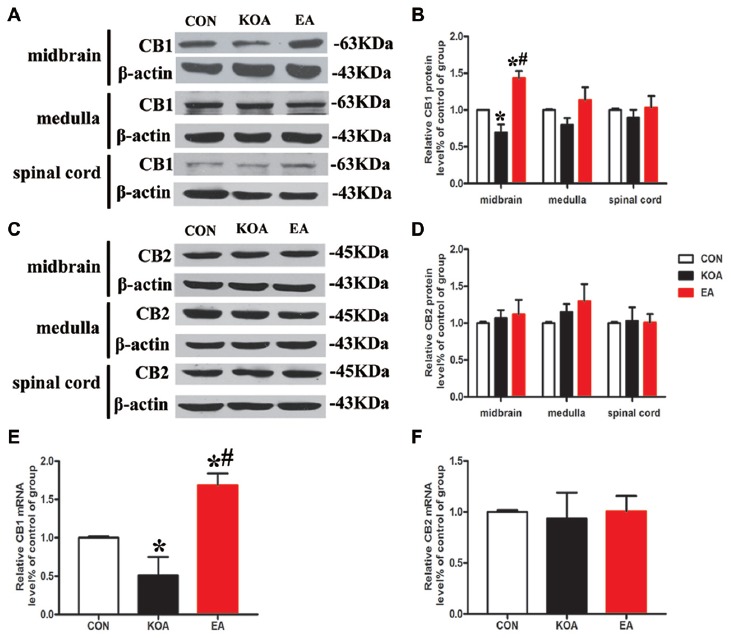
Quantitative analysis of protein and mRNA levels of CB1 and CB2 receptors in the midbrain, medulla and spinal cord tissues. **(A,C)** Representative gel images show the protein level of CB1 and CB2 receptors in the midbrain, medulla and spinal cord tissues obtained from control (CON), KOA, and KOA treated with 2 Hz + 1 mA EA. β-actin was used as a loading control. The protein band at 63 kDa and 45 kDa corresponds to the CB1 and CB2 receptors, respectively. **(B,D)** Summary data show the effect of KOA and EA on the protein level of CB1 and CB2 receptors in the midbrain, medulla and spinal cord tissues. **(E,F)** Effects of KOA and EA on the mRNA level of CB1 and CB2 receptors in the midbrain. Data are expressed as means ± SD (*n* = 6 mice in each group). *p < 0.05 vs. the control group; ^#^*p* < 0.05, compared with the KOA group.

The CB1 and CB2 receptor protein bands were presented in the midbrain tissues (Figures 3A,C). The CB1 receptor protein level in the midbrain was significantly lower than that in the control group 4 weeks after KOA induction (Figure 3B). EA treatment at 2 Hz + 1 mA significantly increased CB1 receptors level in the midbrain compared with that in the KOA group (Figure 3B).

The CB2 receptor protein level in the midbrain did not differ significant among different groups 4 weeks after KOA induction (Figure 3D). Also, the protein levels of CB1 and CB2 receptors in the medulla and spinal cord were similar in all groups 4 weeks after KOA induction (Figures 3B,D).

We also measured the mRNA levels of CB1 and CB2 receptors in the midbrain. The mRNA level of CB1 receptors in the KOA group was significantly lower than that in the control group 4 weeks after KOA induction (Figure 3E). EA treatment at 2 Hz + 1 mA significantly increased the mRNA level of CB1 receptors compared with that in KOA (Figure 3E). The mRNA level of CB2 receptors did not differ significantly among different groups (Figure 3F).

### EA Reverses the Reduction of CB1 Receptors Expression in GABAergic Neurons but Not Glutamatergic Neurons in PAG of KOA Mice

The PAG is a major site of the analgesic actions of μ-opioids and cannabinoids and forms part of a descending analgesic pathway that projects via the RVM to modulate nociceptive transmission within the spinal dorsal horn (Fields et al., 1991). Moreover, CB1 receptors are expressed at the nerve terminals of both GABAergic and glutamatergic neurons in PAG (Tsou et al., 1998; Vaughan et al., 2000).

**Figure 4:**
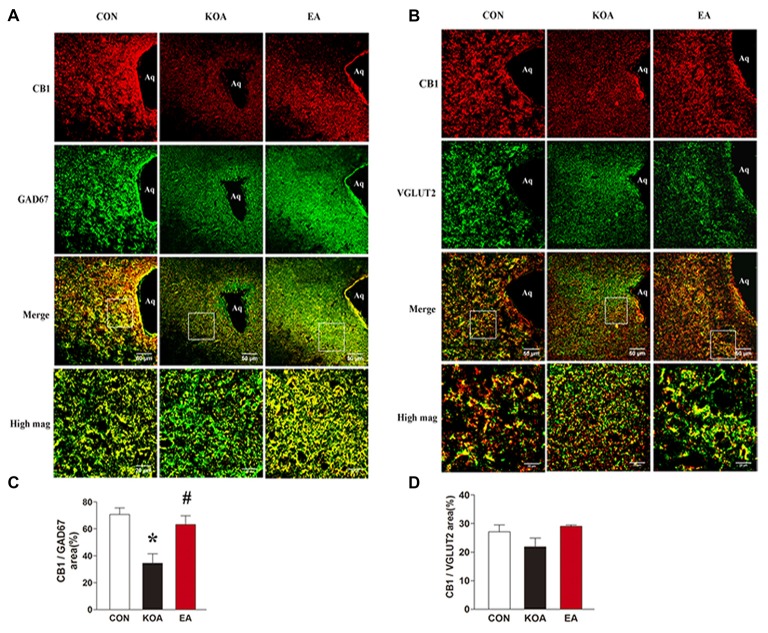
Double immunolabeling of CB1 receptors and the GABAergic neurons marker GAD67 or Glutamatergic neurons marker VGLUT2 in the periaqueductal gray (PAG). **(A)** CB1 receptors labeling (red); GAD67 labeling (green); double-labeled cells (yellow). **(B)** CB1 receptors labeling (red); VGLUT2 labeling (green); double-labeled cells (yellow). Scale bar, 50 μm. Scale bar for high magnification images (high mag), 20 μm. **(C)** Summary data show the percentage of the area of double stained cells in the area of total GAD67-immunoreactive cells. **(D)** Summary data show the percentage of the area of double stained cells in the area of total VGLUT2-immunoreactive cells. Data are expressed as means ± SD (*n* = 6 mice in each group). *p < 0.05, vs. the control group; ^#^*p* < 0.05, compared with the KOA group.

To dissect the roles of CB1 receptors on GABAergic and glutamatergic PAG neurons, we investigated the co-localization between CB1R and GAD67 as well as VGLUT2 immunoreactivities. The percentage of the area of double stained CB1R and GAD67 in the area of total GAD67 immunoreactivity was significantly lower in the KOA group than the control group (Figures 4A,C). Also, 2 Hz + 1 mA EA significantly increased the expression of CB1 receptors on GABAergic neurons (Figures 4A,C). However, EA had no significant effect on the percentage of the area of double stained CB1R and VGLUT2 in the area of total VGLUT2 immunoreactivity of KOA mice (Figures 4B,D). These data suggest that EA significantly reverses the reduction of CB1 receptors expression in GABAergic neurons but not glutamatergic neurons in PAG.

### EA Reverses the Reduction in 2-AG but Not AEA Concentration in the Midbrain of KOA Mice

Endocannabinoid family consists of two classes of well characterized ligands including N-acylethanolamines, such as N-arachidonoyl ethanolamide or anandamide (AEA), and the monoacylglycerols, such as 2-AG (Devane et al., 1992). Endocannabinoids are present in the midbrain, medulla and spinal cord (Herkenham et al., 1991). Moreover, the PAG is a major site of cannabinoid-mediated analgesia in the central nervous system (Vaughan et al., 2000; Wilson et al., 2008). We thus determined whether EA increases the concentration of anandamide and 2-AG concentration in midbrain.

**Figure 5:**
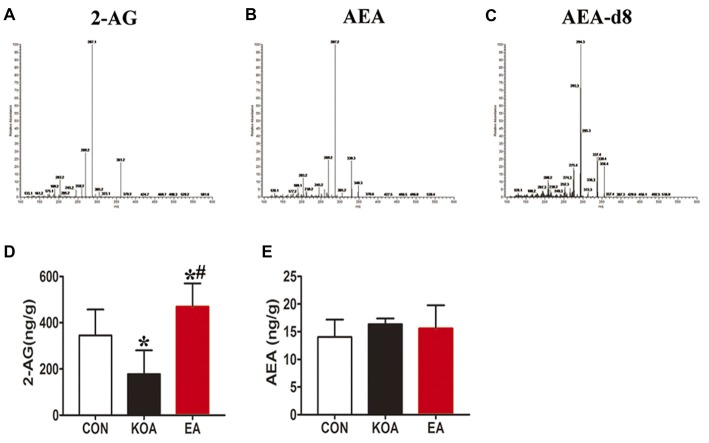
Quantitative analysis of arachidonoylethanolamine (AEA) and 2-arachidonoylglycerol (2-AG) concentrations in the midbrain. **(A–C)** The parent ion and daughter ion mass spectra for 2-AG, AEA and AEA-d8. **(D,E)** Summary data show the effect of KOA and EA on the concentration of 2-AG and AEA in the midbrain. Data are expressed as means ± SD (*n* = 6 mice in each group). *p < 0.05 vs. the control group; ^#^*p* < 0.05, compared with the KOA group.

For this series of experiments, we used three groups of mice: control, KOA, and 2 Hz + 1 mA EA. Endogenous cannabinoids, including 2-AG and AEA were detected in midbrain in all the three groups 4 weeks after KOA induction (Figures 5A,B). Spectra of the parent ion and daughter ion scan for IS AEA-d8 is presented in Figure 5C.

The 2-AG level in the midbrain was significantly lower in the KOA group than the control group (Figure 5D). EA significantly increased the 2-AG level in the midbrain of KOA mice (Figure 5D). However, EA had no significant effect on the anandamide concentration in the midbrain of KOA mice (Figure 5E).

### Microinjection of the CB1 Receptor Antagonist AM251 Into the vlPAG Can Reverse the EA Effect on Pain Hypersensitivity and DNIC Function

Previous studies have demonstrated that stimulating the vlPAG results in antinociception (Behbehani et al., 1990) by activating a descending pain inhibitory pathway. Moreover, cannabinoid system contributes to the antinociception initiated from the vlPAG, which is enriched with cannabinoid receptors (Tsou et al., 1998). Therefore, we microinjected the CB1 receptor antagonist AM251 into the vlPAG to determine whether CB1 receptors contribute to the EA effect on pain hypersensitivity and DNIC function in KOA mice.

**Figure 6:**
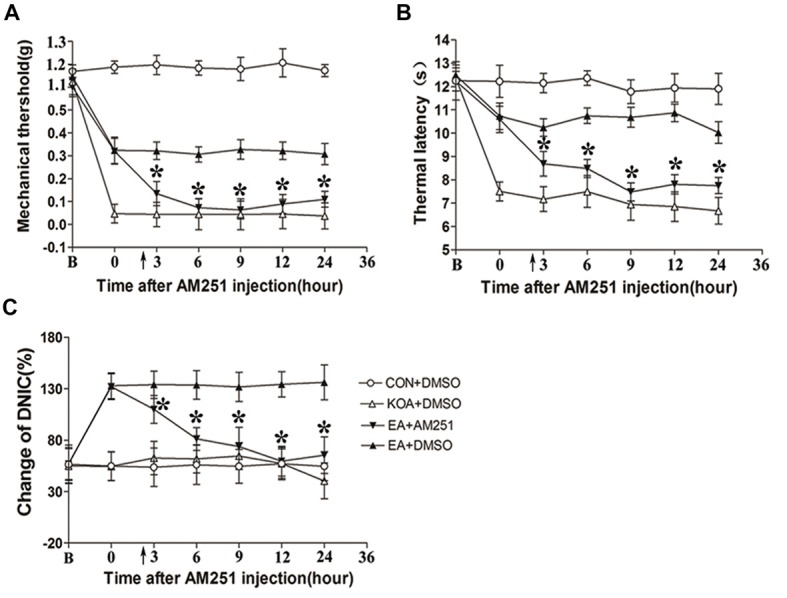
Microinjection of the CB1 receptor antagonist AM251 into the vlPAG can reversed the EA effect on pain hypersensitivity and DNIC function. **(A)** Time course of changes in the tactile threshold in KOA mice after AM251 microinjection. **(B)** Time course of changes in the thermal withdrawal threshold in KOA mice after AM251 microinjection. **(C)** Time course of changes in the DNIC function in KOA mice after AM251 microinjection. EA was administered for 30 min, after 2.5 h AM251 microinjection, as indicated by arrows. B = baseline (The baseline nociceptive thresholds and DNIC function were tested for 3 days before MIA injection, and the mean value was calculated as baseline data); Data are expressed as means ± SD (*n* = 8 mice in each group). *p < 0.05, compared with the EA+DMSO group.

Mice were randomly divided into CON+DMSO, KOA+DMSO, EA+AM251 and EA+DMSO groups. On the 14th day after KOA induction, each mouse in the EA+AM251 group was microinjection with of AM251 (0.1 μl; 1 mg/ml; Tjen et al., 2009) into the vlPAG. We found that microinjection of the CB1 receptor antagonist AM251 but not DMSO into the vlPAG, significantly reversed the EA effect on the tactile withdrawal thresholds within 24 h after injection (Figures 6A,B). AM251 also significantly reversed the EA effect on improve DNIC function of KOA mice within 24 h after injection (Figure 6C).

### CB1 Receptors on GABAergic Neurons Is Involved in the EA Effect on Pain Hypersensitivity and DNIC Function

Previous research has found that CB1 receptors are localized at the axon terminals of GABAergic and glutamatergic PAG neurons (Tsou et al., 1998; Vaughan et al., 2000). Therefore, we determined whether CB1 receptors on GABAergic neurons or glutamatergic neurons contribute to the EA effect on pain hypersensitivity and DNIC function in KOA mice.

**Figure 7:**
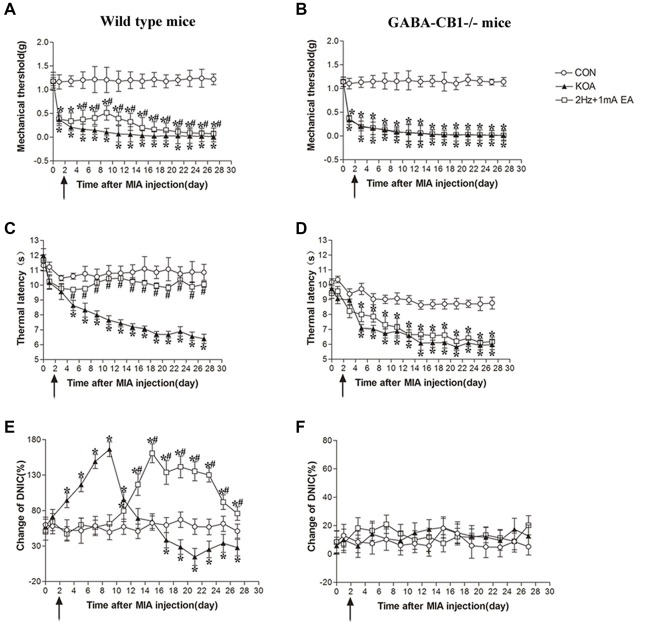
Comparison of the effects of EA on pain hypersensitivity and the DNIC function in wild-type mice and GABA-CB1^−/−^ mice subjected to KOA induction. **(A,B)** Time course of changes in the tactile threshold in wild-type mice and GABA-CB1^−/−^ mice after KOA induction. **(C,D)** Time course of changes in the thermal withdrawal threshold in wild-type mice and GABA-CB1^−/−^ mice after KOA induction. **(E,F)** Effects of KOA and EA on DNIC function of wild-type mice and GABA-CB1^−/−^ mice subjected to KOA induction. EA was administered for 30 min, once every other day for 4 weeks, starting from 2 days after MIA injection, as indicated by arrows. Data are expressed as means ± SD (*n* = 10 mice in each group). *p < 0.05, compared with the control group; ^#^*p* < 0.05, compared with the KOA group.

In wild-type mice, EA significantly increased the thermal latency and tactile withdrawal threshold in KOA mice (Figures 7A,C). In GABA-CB1^−/−^ mice, KOA induction still decreased the thermal latency and tactile threshold. However, EA have no significant effect on thermal hyperalgesia and tactile allodynia in KOA mice (Figures 7B,D).

In wild-type mice, the DNIC function 17 days after KOA induction was significantly lower than that in the vehicle-injected control group (Figure 7E). EA significantly increased the DNIC function 13 days after KOA induction (Figure 7E). In GABA-CB1^−/−^ mice, KOA induction and EA treatment had no effect on the DNIC function of GABA-CB1^−/−^ mice subjected to KOA induction (Figure 7F).

### CB1 Receptors on Glutamatergic Neurons Contribute to the Effect of EA on Pain Hypersensitivity but Not DNIC Function

**Figure 8:**
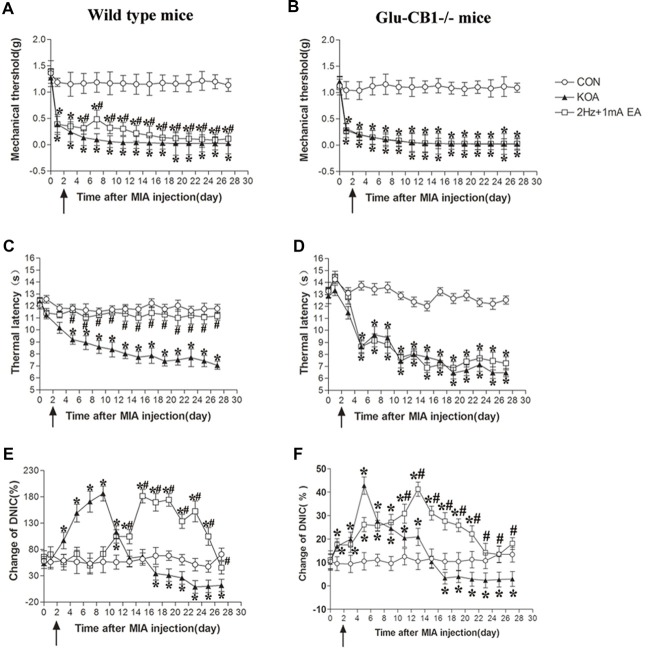
Comparison of the effects of EA on pain hypersensitivity and the DNIC function in wild-type mice and Glu-CB1^−/−^ mice subjected to KOA induction. **(A,B)** Time course of changes in the tactile threshold in wild-type mice and Glu-CB1^−/−^ mice after KOA induction. **(C,D)** Time course of changes in the thermal withdrawal threshold in wild-type mice and Glu-CB1^−/−^ mice after KOA induction. **(E,F)** Effects of KOA and EA on DNIC function of wild-type mice and Glu-CB1^−/−^ mice subjected to KOA induction. EA was administered for 30 min, once every other day for 4 weeks, starting from 2 days after MIA injection, as indicated by arrows. Data are expressed as means ± SD (*n* = 10 mice in each group). *p < 0.05, compared with the control group; ^#^*p* < 0.05, compared with the KOA group.

We used Glu-CB1^−/−^ mice to determine whether CB1 receptors on glutamatergic neurons are involved in the EA effect. In wild-type mice, EA significantly increased the thermal latency and tactile threshold after KOA induction (Figures 8A,C). In Glu-CB1^−/−^ mice, KOA induction still decreased the thermal latency and tactile threshold. However, similar to GABA-CB1^−/−^ mice, EA had no significant effect on the thermal latency and mechanical threshold of Glu-CB1^−/−^ mice subjected to KOA induction (Figures 8B,D).

In wild-type mice, KOA significantly increased the DNIC function from the 3rd to 13th day and decreased the DNIC function17 days after KOA induction, which can be significantly reversed by EA (Figure 8E). In Glu-CB1^−/−^ mice, KOA significantly increased the DNIC function from the 1st to 13th day and decreased DNIC function 17 days after KOA induction (Figure 8E). In Glu-CB1^−/−^ mice, EA still significantly reversed the DNIC function impaired by KOA induction (Figure 8F).

### CB1 Receptors on GABAergic Neurons but Not Glutamatergic Neurons Are Involved in the EA Effect on 5-HT Concentration in the Medulla

In the PAG and RVM, activation of CB1 receptors may directly inhibit transmitter release from the terminals of GABAergic neurons, thus disinhibiting the release of serotonin and norepinephrine from neurons in the medulla (Vaughan et al., 2000; Meng and Johansen, 2004). Fluorescence spectrophotometry has the advantages of high selectivity, high sensitivity, low detection limit, wide application and abundant information. Therefore, we used the fluorescence spectrophotometry to determine whether CB1 receptors are involved in the effect of EA on the 5-HT concentration in the medulla.

**Figure 9:**
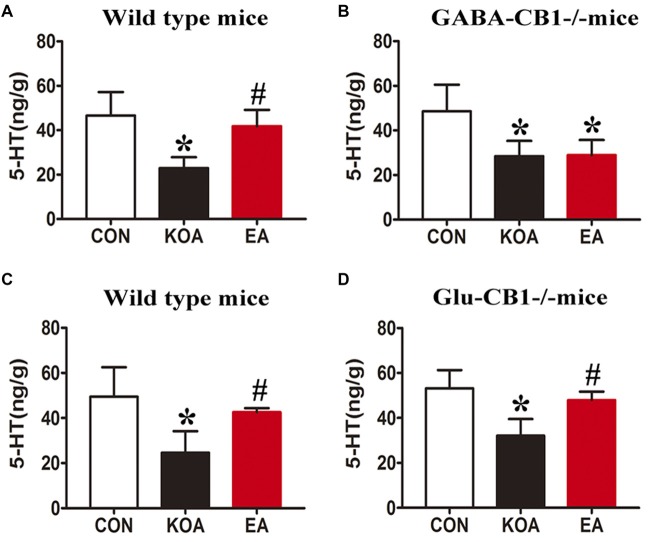
Quantitative analysis of the 5-HT concentration in the medulla. **(A,B)** Summary data show the effect of EA on the 5-HT concentration in the medulla of wild-type mice **(A)** and GABA-CB1^−/−^ mice **(B)**. **(C,D)** Summary data show the effect of EA on the 5-HT concentration in the medulla of wild-type mice **(C)** and Glu-CB1^−/−^ mice. **(D)** Data are expressed as means ± SD (*n* = 6 mice in each group). *p < 0.05, vs. the control group; ^#^*p* < 0.05, compared with the KOA group.

In wild-type mice, KOA induction significantly reduced the 5-HT level in the medulla, and EA treatment significantly reversed the reduction in the 5-HT level caused by KOA induction (Figures 9A,C).

In GABA-CB1^−/−^ mice, KOA induction significantly decreased the 5-HT level in the medulla (Figure 9B). However, EA treatment failed to reverse the reduction in the level of 5-HT caused by KOA induction (Figure 9B).

In Glu-CB1^−/−^ mice, KOA induction also significantly reduced the 5-HT level in the medulla (Figure 9D). EA treatment largely reversed the 5-HT level reduced by KOA induction in Glu-CB1^−/−^ mice (Figure 9D).

## Discussion

Previous studies have shown that the lack of DNIC function is closely associated with chronic pain in patients (Landau et al., 2010; Seifert and Maihofner, 2011). We found in the present study that KOA induction caused a biphasic change in the DNIC function: an initial compensatory increase during acute pain (3–11 days after KOA induction) and a persistent reduction during chronic pain (17 days after KOA induction). Our findings are consistent with previous reports showing that animals subjected to acute inflammatory knee injury exhibit normal DNIC function (Danziger et al., 2001). Impaired DNIC function is present in chronic, but not acute, monoarthritis in the rat (Danziger et al., 1999). DNIC is an endogenous analgesic system likely mediated by the descending inhibitory control from supraspinal structures (Le Bars et al., 1979). The DNIC function was significantly increased in the early stage (3–11 days after KOA induction), since severe acute pain may mobilize the endogenous analgesia system. However, 17 days after KOA induction, the endogenous analgesic transmitters such as endocannabinoid may be exhausted, thus decreasing the DNIC function.

Endocannabinoids within the central nervous system are an important neuromodulatory system involved in the control of pain transmission (Walker and Huang, 2002). Both endocannabinoid ligands and CB receptors are presnt along the nociceptive and decending inhibitory pathways (Mitrirattanakul et al., 2006). In the present study, we showed that the 2-AG level was decreased in the midbrain 4 weeks after KOA induction when the DNIC function was diminished. We also found that the expression of CB1, but not CB2, receptors in the midbrain were decreased 4 weeks after KOA induction, suggesting that diminished DNIC function is associated with reduced cannabinoid ligand and CB1 receptors expression in the midbrain.

CB1 receptors are expressed at the nerve terminals of both GABAergic and glutamatergic neurons in the PAG (Tsou et al., 1998; Vaughan et al., 2000), suggesting that CB1 receptors activation may modulate GABAergic and glutamatergic neurotransmission. We found that KOA induced deficit of the CB1R expression on GABAergic but not glutamatergic neurons. In line with this result, the initial increase in DNIC function caused by KOA induction was abolished by conditional knockout of CB1 receptors on GABAergic but not glutamatergic neurons. Importantly, the reduced DNIC function during the chronic pain after KOA induction was observed in mice with conditional knockout of CB1 receptors on glutamatergic but not GABAergic neurons. These findings suggest that CB1 receptors on GABAergic but not glutamatergic neurons play a critical role in regulating the DNIC function and inhibiting the chronic pain in this KOA animal model. Nevertheless, the sites of CB1 receptors in the nervous system are unclear, and it is possible that CB1 receptors on GABAergic and glutamatergic neurons regulate ascending nociceptive and descending inhibitory pathways through different mechanisms (Samineni et al., 2017).

Previous studies have demonstrated that CB1 receptors-mediated analgesia results from inhibition of GABA release from PAG neurons and subsequent disinhibition of the descending inhibitory pathway involving 5-HT (Meng et al., 1998; Vaughan et al., 2000). We found that KOA induction significantly reduced the 5-HT level in the medulla. Furthermore, a role for descending noradrenergic and/or serotonergic inhibitory pathways has been demonstrated using behavioral (Kimura et al., 2015; Ito et al., 2018) and electrophysiological studies (Bannister et al., 2015, 2017) in spinal nerve ligation (SNL) model. We also found that the baseline of DNIC function of GABA-CB1^−/−^ and Glu-CB1^−/−^mice was decreased compared with that in the wild-type mice, but 5-HT level is normal. Cannabinoids are thought to produce analgesic actions in the PAG and RVM by reducing GABAergic inhibition of release of 5-HT from output neurons (Vaughan et al., 2000). Since CB1 receptors may not affect the synthesis of 5-HT, 5-HT level is normal in GABA-CB1^−/−^ and Glu-CB1^−/−^mice. However, when DNIC is evaluated in GABA-CB1^−/−^ and Glu-CB1^−/−^ mice, the release of 5-HT may be over-inhibited by GABA, thus decreasing DNIC function in GABA-CB1^−/−^ and Glu-CB1^−/−^mice.

We have demonstrated that EA significantly increases the AEA level and CB2 receptor activity in the periphery (Chen et al., 2009; Zhang et al., 2010). In the present study, we provide new evidence that the low frequency and high intensity (2 Hz + 1 mA) of EA reversed the impaired DNIC function and inhibiting chronic pain of KOA. We found that EA reversed the reduced expression of CB1 receptors and the level of 2-AG in the midbrain during chronic pain of KOA. Thus, EA may restore the impaired endogenous cannabinoid system during chronic pain and potentiate DNIC function. Consistently, knockout of CB1 receptors on GABAergic, but not glutamatergic neurons blocked the effect of EA on enhancing DNIC function and 5-HT level in the medulla reduced by KOA induction, suggesting that CB1 receptors on GABAergic neurons are involved in the disinhibition of 5-HT-related descending pathway and the EA effect on restoring the DNIC function during chronic pain of KOA. However, conditional knockout of CB1 receptors on both GABAergic or and glutamatergic neurons blocked analgesic effect of EA, it is possible that CB1 receptors on glutamatergic neurons inhibit ascending nociceptive pathways through different mechanisms.

Deletion of CB1R gene from GABA-CB1R-KO or Glu-CB1R-KO mice obtained by intraperitoneal injections of tamoxifen, which may lead to the lack of CB1 expression in GABAergic neurons or glutamatergic neurons in a variety of nerve tissues, rather than only in the midbrain. It seemed that EA improving DNIC function is not only achieved through the regulation of the midbrain. However, in wild-type mice, we found that EA reversed the reduced expression of CB1 receptors only in the midbrain but not in medulla and spinal cord. In addition, microinjection of the CB1 receptor antagonist AM251 into the vlPAG can reversed the EA effect on pain hypersensitivity and DNIC function. Previous study has demonstrated that c-Fos expression in the PAG is induced by EA and the PAG neurons activated by EA might be the GABAergic neurons (Fusumada et al., 2007). These findings suggested that EA mainly potentiate the expression of CB1 receptors in the midbrain and enhance the DNIC function by activating CB1 receptors in vlPAG.

In conclusion, our study provides new information about the important role of the cannabinoid system in regulating the DNIC function during chronic pain in a KOA mouse model. EA may potentiate the endogenous cannabinoid system and the expression of CB1 receptors on GABAergic neurons in the midbrain to enhance the 5-HT related descending inhibitory control and DNIC function during chronic pain. Thus, our results suggest that the endocannabinoid (2-AG)-CB1R-GABA-5-HT is a novel signaling pathway involved in the EA improving DNIC function, thus inhibiting chronic pain in KOA mouse model.

Our findings may help to identify new strategies to optimize parameters of EA and much effectively exert therapeutic effect of inhibiting chronic pain.

## Author Contributions

ML, QW and H-LP conceived and designed the experiments. X-CY did most of the experiments and analyzed the data; C-HW, FG and H-PL helped with the behavior test experiments. HZ helped with the intra-vlPAG microinjection experiments. BZ and H-CX helped with the 5-HT test experiments. BZ, X-HJ and WH helped with analyzing the data. L-ZX provided the GABA-CB1^−/−^ and Glu-CB1^−/−^ mice. C-YL helped with feeding the animals. X-CY, H-LP and ML wrote the manuscript. All authors reviewed the manuscript.

## Conflict of Interest Statement

The authors declare that the research was conducted in the absence of any commercial or financial relationships that could be construed as a potential conflict of interest.

## Supplementary Material

FIGURE S1 A schematic diagram shows the points used in the study for the treatment of KOA. Two acupuncture needles were inserted into two acupoints corresponding to Ex-LE4 and ST35 in humans, as indicated by arrows. Ex-LE4 is located at the medial cavity of the patella and the patellar ligament, and ST35 lies on the lateral cavity of the patella and patellar ligament.
