# Supplementary figures and images for: Electroacupuncture Potentiates Cannabinoid Receptor-Mediated Descending Inhibitory Control in a Mouse Model of Knee Osteoarthritis
Source: Front Mol Neurosci. 2018 Apr 6;11:112. doi: 10.3389/fnmol.2018.00112 (PMC5897736; doi:10.3389/fnmol.2018.00112)

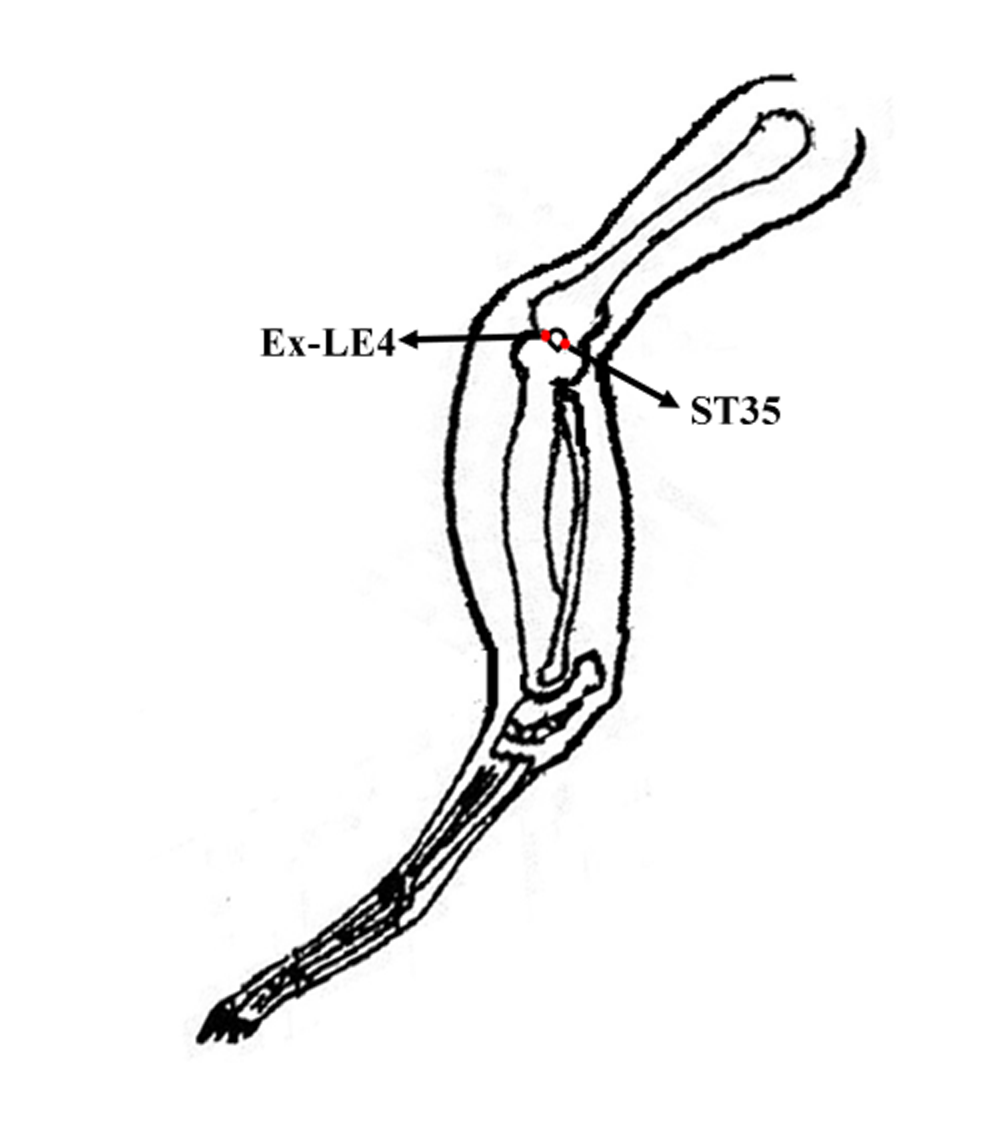

Supplement: FIGURE S1 — A schematic diagram shows the points used in the study for the treatment of KOA. Two acupuncture needles were inserted into two acupoints corresponding to Ex-LE4 and ST35 in humans, as indicated by arrows. Ex-LE4 is located at the medial cavity of the patella and the patellar ligament, and ST35 lies on the lateral cavity of the patella and patellar ligament. [file Image_1.TIF]
